# Intraoperative 3D Tomosynthesis of the Elbow in a Simulated Prone-Position Setup: A Cadaveric Study

**DOI:** 10.3390/diagnostics16193178

**Published:** 2026-09-30

**Authors:** Benno Bullert, Livia Morlock, Fenna Brunken, Jula Gierse, Paul A. Gruetzner, Sven Y. Vetter, Nils Beisemann

**Affiliations:** BG Klinik Ludwigshafen, Department for Orthopaedics and Trauma Surgery at Heidelberg University, 67071 Ludwigshafen, Germany

**Keywords:** intraoperative 3D imaging, CBCT, tomosynthesis, C-arm, elbow surgery, distal humerus fracture

## Abstract

**Background/Objectives**: Fractures of the distal humerus and olecranon often require surgical intervention due to their complexity and intra-articular fracture pattern. Precise implant positioning and articular surface reconstruction are crucial for optimal outcomes. Intraoperative 3D imaging offers advantages over 2D fluoroscopy by providing enhanced visualization of fracture reduction and implant alignment. However, limited space in the prone position restricts the use of standard 3D imaging with complete orbital rotation. This cadaver study investigates the feasibility and intra-articular screw detection performance of intraoperative 3D tomosynthesis with reduced orbital rotation at the elbow. **Methods**: In this cadaver study, six distal humeri and six olecranons were examined using an isocentric 3D C-arm with a complete 200° acquisition. The disarticulated upper extremities were positioned to reproduce the prone surgical setup. Reduced-angle datasets ranging from 200° to 100° were reconstructed retrospectively from the complete acquisition, with and without metal artifact reduction (MAR). Double-plate osteosynthesis was applied to the distal humerus, while an anatomic olecranon plate was used for fixation on the olecranon. To evaluate detection capabilities, screw positions were deliberately modified to include both correct placements and intra-articular malpositions. Image quality (5-point Likert scale) and certainty of intra-articular screw assessment were evaluated by three blinded raters; sensitivity and specificity were calculated relative to the complete 200° imaging-based reference condition. **Results**: Image quality, sensitivity, and specificity decreased with reduced orbital rotation. MAR significantly improved image quality and specificity, but not sensitivity. At an orbital rotation of 160°, MAR-reconstructed tomosynthesis achieved acceptable image quality (3.36 ± 0.53), high sensitivity (94.9%), and high specificity (93.9%) for detecting intra-articular screws. **Conclusions**: Intraoperative tomosynthesis with MAR provides acceptable image quality and high performance for intra-articular screw detection at the elbow under the present experimental conditions. This approach may support intraoperative assessment in prone-position elbow surgery when full orbital rotation is not feasible. Clinical studies are needed to confirm its value in routine practice.

## 1. Introduction

Fractures of the distal humerus and proximal forearm account for 5.5% of adult fractures [1]. Both olecranon fractures and distal humerus fractures are usually treated surgically [2,3]. The treatment of distal humerus fractures is particularly challenging because of the complex anatomy [4].

In fracture surgery, accurate implant positioning and restoration of the articular surface are important for achieving optimal outcomes [5]. Because of curved articular surfaces and complex three-dimensional anatomy, conventional 2D fluoroscopy may provide insufficient information on fracture reduction and implant position [6,7,8]. Several studies have demonstrated the advantages of intraoperative 3D imaging across various anatomical regions for assessing implant malposition and malreduction compared with 2D fluoroscopy [8,9,10,11,12,13,14,15,16,17].

Despite the elbow’s complex anatomy, only a few studies have examined preoperative 3D imaging [18,19,20]. Preoperative 3D imaging has been shown to be superior to conventional 2D radiographs in detecting fractures and intra-articular and multi-fragmentary fracture patterns [18]. To our knowledge, only one clinical study has specifically evaluated intraoperative 3D imaging in elbow fracture surgery. This study reported additional information on screw malposition and malreduction that resulted in immediate correction in 16.7% of cases, particularly in complex intra-articular distal humerus fractures [21].

Due to limited space in the prone position—the most common position for surgical access to the olecranon and distal humerus [22,23]—conventional intraoperative 3D imaging with a complete orbital rotation is often not feasible. To accommodate this spatial limitation, the orbital rotation must be reduced; however, this reduction prevents complete data acquisition, resulting in a transition from true 3D imaging to tomosynthesis [24]. Tomosynthesis generates a three-dimensional dataset from a limited number of projections and is primarily used in mammography, chest imaging, and radiotherapy [25].

To date, the impact of reduced orbital rotation during intraoperative 3D imaging of the elbow has not been systematically investigated. This cadaver study therefore evaluates intraoperative tomosynthesis of the elbow under realistic spatial constraints. Based on preliminary positioning tests performed in the prone position using the investigated C-arm and surgical setup, an orbital rotation of approximately 160° was achievable without collision. The study therefore aimed to determine whether intraoperative tomosynthesis provides sufficient image quality and enables reliable detection of implant malposition at the distal humerus and the olecranon.

## 2. Materials and Methods

The study was approved by the institutional ethics committee (application number: 2024-17547; date: 7 May 2024).

An isocentric 3D C-arm (Cios Spin, Siemens Healthineers AG, Erlangen, Germany) was used for this study. During a 200° orbital rotation, the system acquired up to 400 projection images for reconstruction of a 3D dataset. The C-arm enabled flexible adjustment of orbital rotation during 3D scan acquisition, ranging from 120 to 200°.

### 2.1. Cadaver and Experimental Setup

Three human cadavers were used for the experiments (6 distal humeri and 6 olecranons). No a priori sample size calculation was performed, as no prior systematic data on the effect of progressively reduced orbital rotation on intraoperative 3D image quality and screw detection at the elbow were available to derive a reliable effect size. To reproduce the intraoperative prone-position setup while allowing a complete 200° acquisition, the upper extremities were disarticulated and secured to an arm table with the elbow flexed to 90°. The C-arm was positioned perpendicular (in the longitudinal axis of the patient) to the experimental setup according to our standard intraoperative positioning.

First, a 3D scan with full orbital rotation (200°) was performed without a metallic implant.

No fracture model was created. A double-plate osteosynthesis (VA-LCP Distal Humeral Plate 2.7/3.5 mm dorsolateral + medial, stainless steel, DePuy Synthes, Raynham, MA, USA) was performed on the distal humerus via the dorsal approach. For the olecranon, osteosynthesis was performed using an anatomic olecranon plate (VA-LCP Olecranon Plate 2.7/3.5 mm, stainless steel, DePuy Synthes, Raynham, MA, USA). For each osteosynthesis, correct screw position (screws not penetrating the articular surface) and screw malposition (screws penetrating the articular surface) were configured. Both screw placements were positioned as close as possible to the articular surface while maintaining a clear distinction between intra- and extra-articular positioning. In cases with intra-articular screw placement, 1–2 screws per dataset perforated the articular surface.

The intended intra- and extra-articular screw positions were independently assessed by three surgeons using the complete 200° 3D dataset, which served as the imaging-based reference condition. If disagreement occurred, the respective screw was repositioned and imaging was repeated until consensus was reached.

### 2.2. Systematic Reduction in Orbital Rotation

A 3D scan with 200° of orbital rotation was used as the base dataset. In the investigated prone-position experimental setup, orbital rotation from 100° to −60° (160° of orbital rotation) could be performed without collision (Figure 1).

At higher orbital rotations, the detector collides with the patient’s back or the operating table. Accordingly, an asymmetric reduction was performed from 200° to 160° orbital rotation (20° steps on the negative end of the orbital rotation), and a symmetric reduction was performed from 160° to 100° orbital rotation (10° steps on both ends of the orbital rotation; Table 1; Figure 2).

To systematically reduce orbital rotation, reduced-angle datasets were reconstructed retrospectively from the projection images of the complete 200° acquisition using the predefined start and end positions shown in Table 1.

Metal artifact reduction (MAR) was additionally applied for each reconstruction. All scans performed are shown in Table 2.

### 2.3. Evaluation of the 3D Datasets

Three raters (two trauma surgery residents and one medical student) independently evaluated the 3D datasets in blinded and randomized order. Image quality was assessed using a structured, study-specific questionnaire based on previously published subjective image quality criteria described by Stübig et al. [26]. The questionnaire comprises ratings of cortical and trabecular bone visualization, articular surface visualization, image artifacts, and overall clinical assessability using a 5-point Likert scale.

In addition, raters assessed the presence or absence of an intra-articular screw and rated their certainty regarding this assessment. For sensitivity and specificity analyses, each rater’s binary screw-position assessment was compared with the imaging-based reference condition and included as an individual rater-level observation. The complete questionnaire is provided in the Appendix A.

### 2.4. Statistics

Statistical analyses were performed with IBM SPSS Statistics Version 29 (IBM Corp., Armonk, NY, USA). Quantitative data are shown as mean ± standard deviation (SD). A significance level of *p* < 0.05 was set. The data comprised repeated measurements obtained under different orbital rotation and reconstruction conditions within the same specimens and experimental configurations. Comparisons across orbital rotation settings were therefore treated as paired, non-normally distributed data. Differences across more than two related conditions were analyzed using the Friedman test, followed by Dunn–Bonferroni-adjusted pairwise post hoc comparisons. The significance level for post hoc comparisons was adjusted for multiple testing.

95% confidence intervals (95% CI) for sensitivity and specificity were calculated using the Clopper–Pearson method.

The “cut-off” values indicate the orbital rotation from which there was no further significant improvement in the corresponding parameter.

The Wilcoxon signed-rank test was used for comparisons between two paired, non-normally distributed conditions. Effect sizes were calculated for the corresponding comparisons and interpreted according to Cohen [27].

Inter-rater reliability was assessed using a two-way mixed-effects, consistency, single-measure intraclass correlation coefficient with 95% CI. The ICC was calculated using R Version 4.4.2 (R Foundation for Statistical Computing, Vienna, Austria). The interpretation of the ICC value follows Cicchetti’s recommendations [28].

## 3. Results

### 3.1. Inter-Rater Reliability

The data showed good inter-rater reliability (ICC = 0.854, 95% CI: 0.846–0.862). Given the strong agreement among raters, the mean rating was used in the analysis. Binary screw-position assessments were analyzed at the individual rater level.

### 3.2. Subjective Image Quality (IQ)

Subjective image quality decreased significantly with decreasing orbital rotation (*p* < 0.001). This decrease was significant for both the distal humerus and the olecranon (both *p* < 0.001; Figure 3).

Post hoc comparisons showed significant differences between all consecutive orbital rotation settings (*p* ≤ 0.009), except between 200° and 180° (*p* = 1.000). Pairwise effect sizes were small (0.05 < r < 0.08), whereas the overall effect of reducing orbital rotation was moderate (r = 0.30).

#### Influence of Metal Implant and Metal Artifact Reduction (MAR) on IQ

IQ decreases with decreasing orbital rotation both with and without metal implant and MAR (all *p* < 0.001) (Figure 4).

IQ is significantly influenced by the presence of metal implants and by reconstruction ± MAR. Post hoc tests show that IQ decreases significantly for orbital rotations below 160° in metal-free datasets (*p* < 0.001). In contrast, a significant decrease is observed for rotations below 180° in the presence of metallic implants (both *p* < 0.001).

Without a metal implant, IQ averages 4.07 ± 0.96 (averaged over orbital rotations of 100–200°). With standard reconstruction, the presence of a metal implant significantly reduces the average to 1.97 ± 0.83 (*p* < 0.001). However, reconstruction with MAR algorithms can significantly improve image quality to an average of 2.98 ± 0.99 in the presence of metallic implants (*p* < 0.001).

### 3.3. Detection of Intra-Articular Screws

#### 3.3.1. Sensitivity and Specificity Relative to the 200° Imaging-Based Reference Condition

Each imaging condition contained 13 reference-positive and 11 reference-negative screw-position observations. Each observation was evaluated by three raters, resulting in 39 positive and 33 negative rater-level assessments for the sensitivity and specificity calculations, respectively.

Reducing orbital rotation resulted in a significant decrease in sensitivity for detecting intra-articular screw placement (*p* < 0.001; Figure 5). Sensitivity increased with increasing orbital rotation and reached a plateau at orbital rotations of approximately 160°, with no further significant improvement at higher orbital rotation angles in the post hoc analysis.

Specificity was also significantly affected by orbital rotation (*p* < 0.001). Post hoc testing revealed a significant reduction in specificity at lower orbital rotations, particularly at rotations < 160°, whereas higher orbital rotations demonstrated consistently high specificity.

At 160° with MAR, 37 of 39 reference-positive rater-level assessments were correctly classified, corresponding to a sensitivity of 94.9% (95% CI: 82.7–99.4%). Of 33 reference-negative rater-level assessments, 31 were correctly classified, corresponding to a specificity of 93.9% (95% CI: 79.8–99.3%). Detailed estimates for all orbital rotations and reconstruction conditions are provided in the Appendix A.

#### 3.3.2. Influence of MAR on Sensitivity and Specificity

The application of metal artifact reduction (MAR) did not result in a statistically significant improvement in sensitivity compared with standard reconstruction (*p* = 0.064; Figure 6A). In contrast, MAR significantly improved specificity across orbital rotation angles (*p* < 0.001; Figure 6B), particularly at lower orbital rotations.

#### 3.3.3. Certainty in the Assessment of Intra-Articular Screws

Diagnostic certainty decreased significantly with decreasing orbital rotation (*p* < 0.001), whereas MAR significantly increased certainty in screw-position assessment (*p* < 0.001).

No significant improvement in diagnostic certainty was observed at orbital rotations above 160° compared with the complete 200° acquisition.

### 3.4. Assessability of the Articular Surfaces

Assessability of the articular surfaces decreased significantly with decreasing orbital rotation (*p* < 0.001) (Figure 7).

Post hoc tests show that with metallic implants, assessability of the articular surface starts to degrade significantly at orbital rotations below 180°. Without metallic implants, this value drops to below 160° (Table 3).

#### Influence of MAR on the Assessability of the Articular Surface

Reconstruction with MAR algorithms significantly improves the assessability of the articular surfaces (all *p* < 0.001). At the distal humerus, a significantly better assessability of the radial articular surface compared to the ulnar articular surface is shown (*p* < 0.001).

## 4. Discussion

The results of this cadaver study demonstrate that intraoperative tomosynthesis of the elbow in the prone position, using an orbital rotation of 160°, provides acceptable subjective image quality. Image quality can be further improved with software-based metal artifact reduction (MAR). Importantly, high sensitivity for detecting intra-articular screws can already be achieved with standard reconstruction, while MAR primarily improves overall image quality and specificity. Visual examples of the different anatomies at decreasing orbital rotations are shown in Figure 8.

### 4.1. Image Quality (IQ)

Reducing orbital rotation decreases the angular information available for reconstruction and consequently reduces subjective image quality. In the present study, image quality declined progressively between 180° and 120°.

Limited-angle tomosynthesis produces plane-dependent image degradation. Slices oriented parallel to the detector at the apex of the acquisition arc retain the highest image quality, whereas orthogonal planes are more affected by blurring and distortion [29].

The acquisition arcs and specimen positioning in the present study were selected to reproduce the spatial constraints encountered during prone-position elbow surgery (Figure 1).

Previous studies have shown that MAR can improve both objective and subjective image quality in intraoperative 3D imaging [30,31]. Similar benefits have been reported for tomosynthesis datasets [32,33].

### 4.2. Detection of Intra-Articular Screws

The detection of implant malposition and immediate intraoperative revision are among the main purposes of intraoperative 3D imaging. In the present study, high sensitivity and specificity relative to the 200° imaging-based reference condition were maintained at an orbital rotation of 160°. In addition, the certainty of screw-position assessment decreased with decreasing orbital rotation, whereas MAR significantly increased diagnostic certainty.

Across extremity trauma surgery, intraoperative 3D imaging provides additional information on implant position compared with conventional fluoroscopy and enables immediate correction of detected malpositions [34,35]. More specifically, at the elbow, Schnetzke et al. reported pathological findings on intraoperative 3D imaging that had not been detected by conventional 2D fluoroscopy in one third of 36 procedures. Immediate intraoperative revision was required in 16.7% of cases, including three previously unrecognized intra-articular screws [21]. In a cadaveric distal humerus fracture model, intra-articular screw placement was correctly assessed in 84% of cases using intraoperative 3D imaging, without a significant difference compared with intraoperative CT [36].

Given the only minor reduction in sensitivity, specificity, and image quality at 160° compared with full orbital rotation, the performance of intraoperative 3D imaging for screw assessment appears to be largely maintained with moderately reduced orbital rotation. This is particularly relevant in prone-position elbow surgery, where complete orbital rotation may be restricted by collisions with the patient or operating table.

### 4.3. Assessment of the Articular Surface

The assessability of the articular surfaces of the elbow depends on the implants, the amount of orbital rotation, and the application of MAR algorithms.

The clinical relevance of articular surface visualization has been demonstrated in elbow surgery, where intraoperative 3D imaging identified residual intra-articular step-offs > 2 mm requiring immediate correction [21]. In an experimental distal humerus fracture model, fluoroscopic 3D imaging also enabled assessment of articular step-offs, although subtle 1 mm incongruities remained difficult to detect [36].

The assessability of the articular surface in the present study represents an indirect parameter, as no fracture or malreduction model was included. Nevertheless, preserved assessability at reduced orbital rotations indicates that visualization of the joint surface remains possible under these acquisition conditions.

In double-plate osteosynthesis on the distal humerus, the humeroulnar joint is more difficult to assess than the humeroradial joint. This may be related to the positioning of the plates on both sides of the humeroulnar articulation, increasing the amount of potentially artifact-generating metal in this region. This interpretation is consistent with previous observations that double plating of the distal humerus combined with olecranon fixation results in a large amount of potentially artifact-generating osteosynthesis material [36].

### 4.4. Clinical Relevance

For an orbital rotation of 160°, scores ranging from acceptable to good were obtained across all evaluated categories. Image quality with MAR was 3.36 ± 0.53; sensitivity and specificity relative to the 200° imaging-based reference condition were 94.9% and 93.9%, respectively; and assessability of the articular surface with MAR was 3.31 ± 0.67.

Intraoperative tomosynthesis may be particularly useful at the distal humerus because of its complex articular anatomy, frequent intra-articular fracture patterns [37], and the need for precise implant positioning and reconstruction [38,39]. Limited access to standard acquisitions with complete orbital rotation has likely contributed to the limited adoption of intraoperative 3D imaging for these fractures.

### 4.5. Limitations

Advanced reconstruction and post-reconstruction algorithms may reduce artifacts associated with limited-angle data acquisition [40]. In this study, good results were already achieved with the standard filtered back-projection. Further studies are necessary to investigate the influence of special reconstructions on the image quality of 3D scans with reduced orbital rotation.

In addition to image quality, the assessability of the reduction is also of great importance. The evaluation of the articular surface was therefore included in the image quality analysis. However, as no articular malreduction was simulated, the direct applicability of the results to the assessment of articular surface reduction may be limited.

Data on the distal humerus were collected using 90° double plating. Transferability to 180° double plating, which is also frequently used, may be limited.

The limited number of cadaveric specimens may limit the generalizability of the findings. Datasets at different orbital rotations were reconstructed from the same specimens and source acquisitions and therefore represent repeated, non-independent observations. Furthermore, screw position was referenced to consensus assessment of the complete 200° 3D dataset by three surgeons rather than direct anatomical verification. Sensitivity and specificity should therefore be interpreted relative to this imaging-based reference condition.

## 5. Conclusions

This cadaveric study demonstrates that intraoperative tomosynthesis of the elbow using a conventional isocentric 3D C-arm can provide acceptable subjective image quality, particularly when combined with metal artifact reduction (MAR) algorithms. Although reduced orbital rotation decreases subjective image quality, under the present experimental conditions, high sensitivity and specificity relative to the 200° imaging-based reference condition for detecting intra-articular screw malposition were maintained at an orbital rotation of 160°.

These findings suggest that reduced-angle intraoperative tomosynthesis may be a practical imaging option for elbow surgery in the prone position when full orbital rotation is not feasible. However, as no fracture or articular malreduction model was included, the ability of tomosynthesis to detect articular surface malreduction remains unknown. The clinical relevance of this technique and its impact on intraoperative decision-making require confirmation in clinical studies.

## Figures and Tables

**Figure 1:**
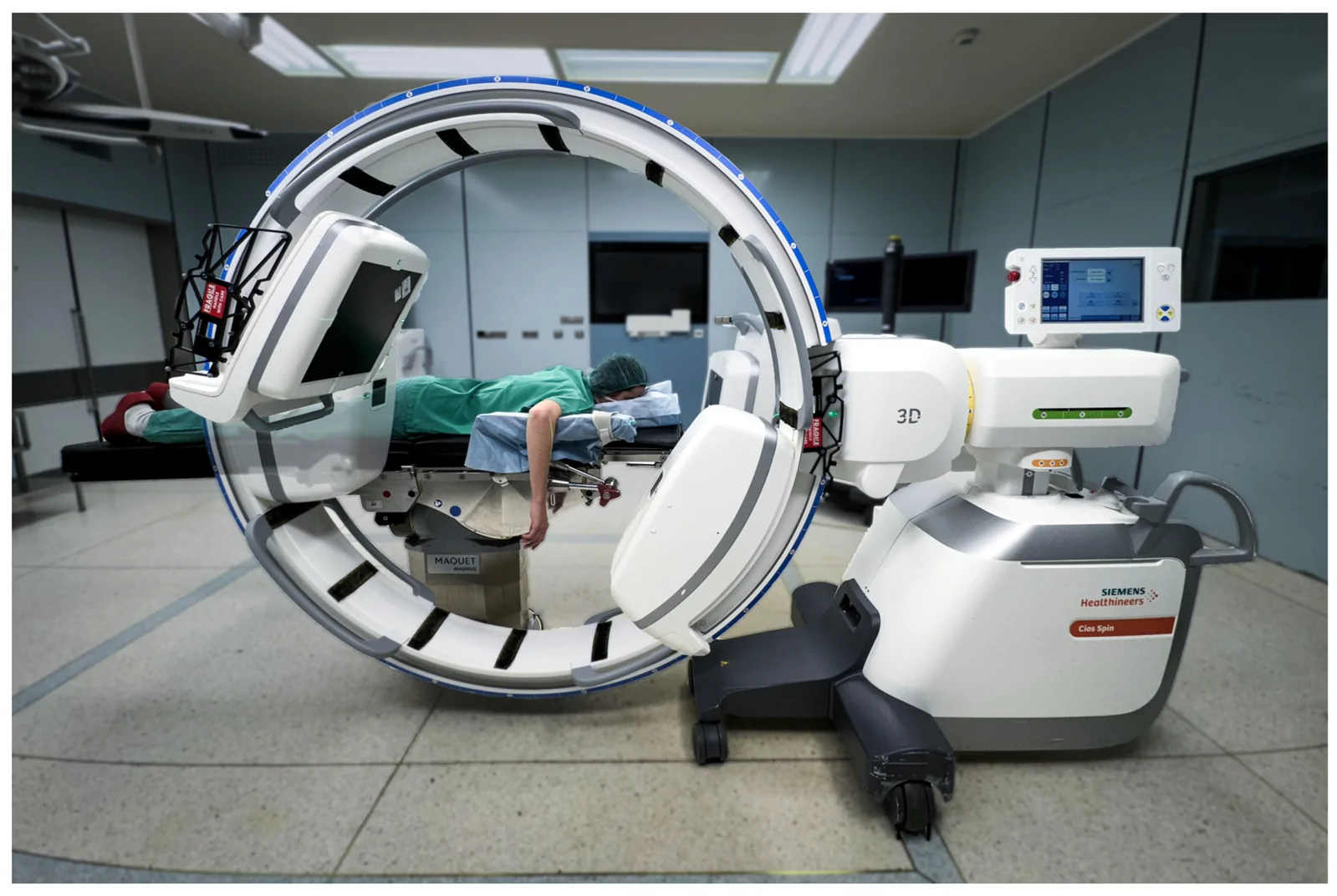
Orbital rotation of 160° in the investigated prone-position experimental setup.

**Figure 2:**
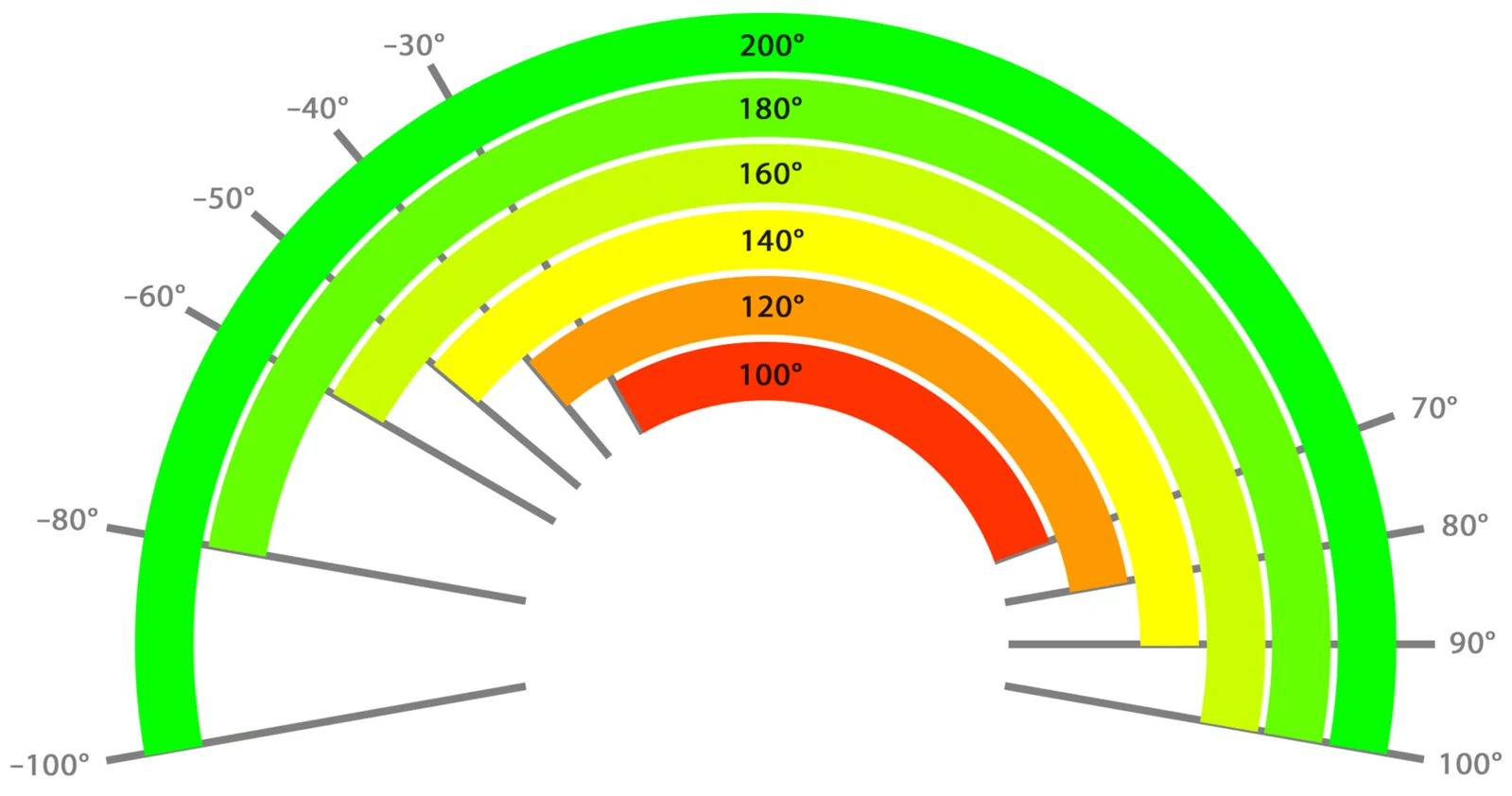
Reduction in Orbital Rotation—Detector Path.

**Figure 3:**
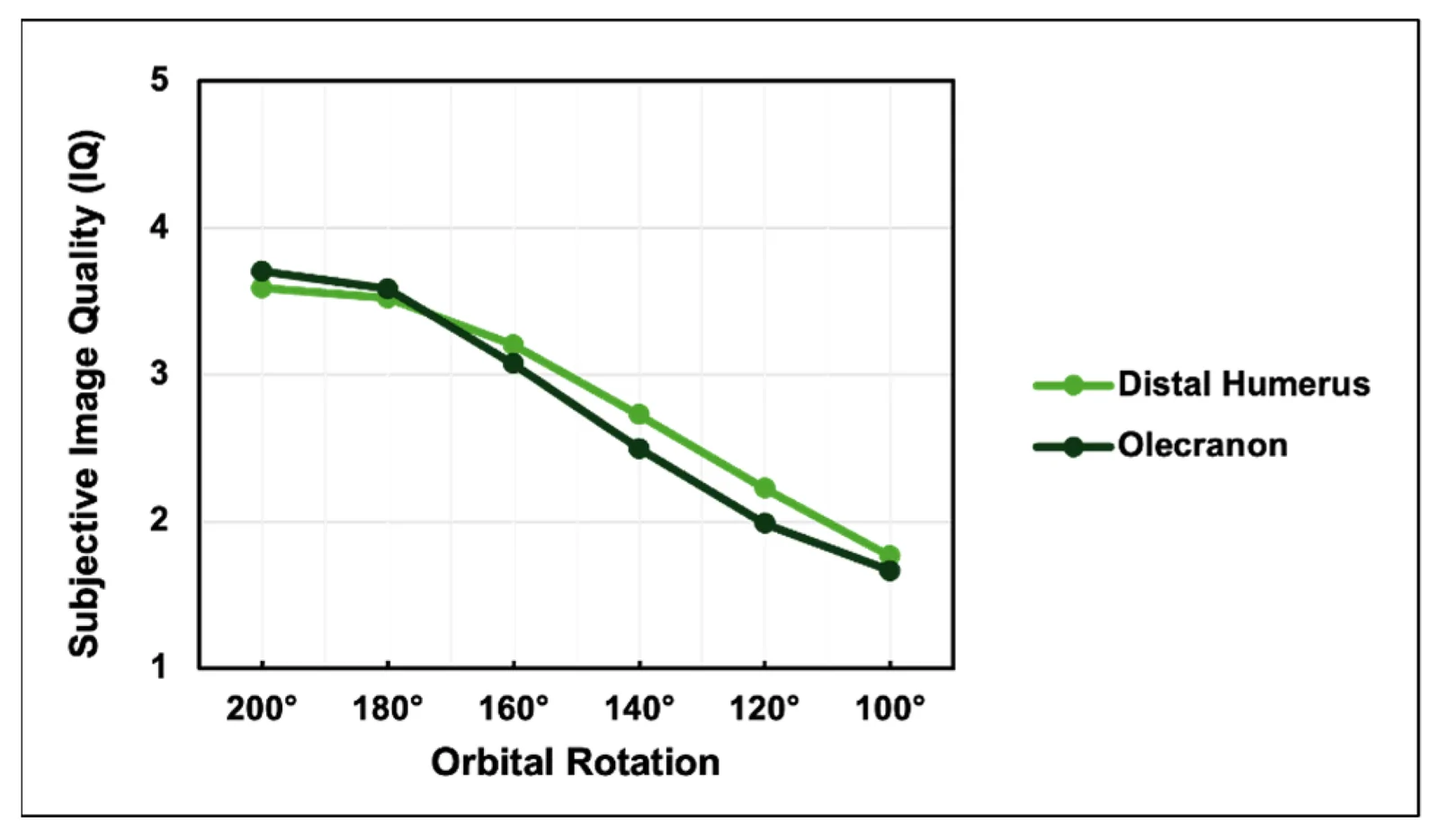
Subjective Image Quality vs. Orbital Rotation.

**Figure 4:**
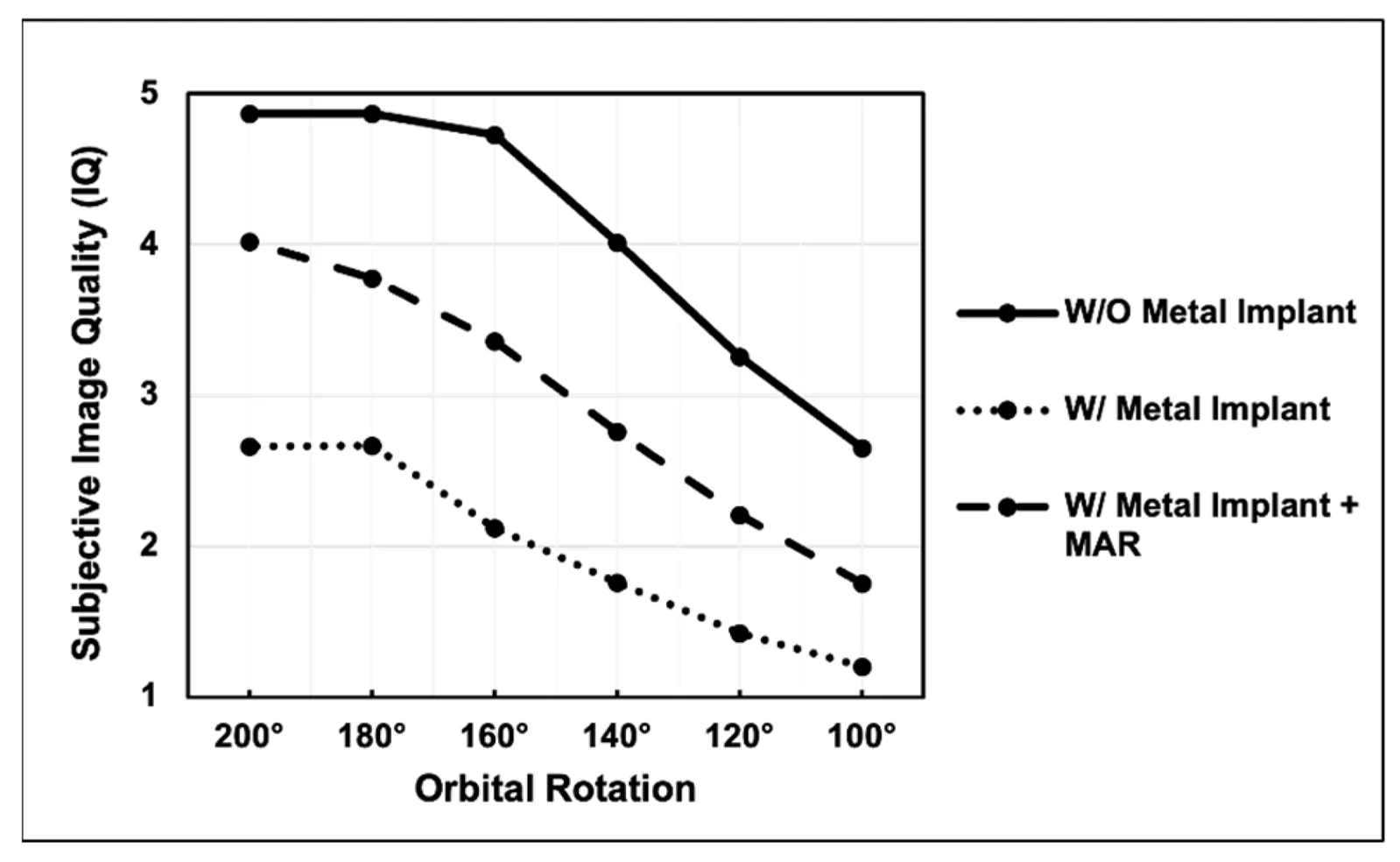
IQ ± Metal Implant/MAR vs. Orbital Rotation.

**Figure 5:**
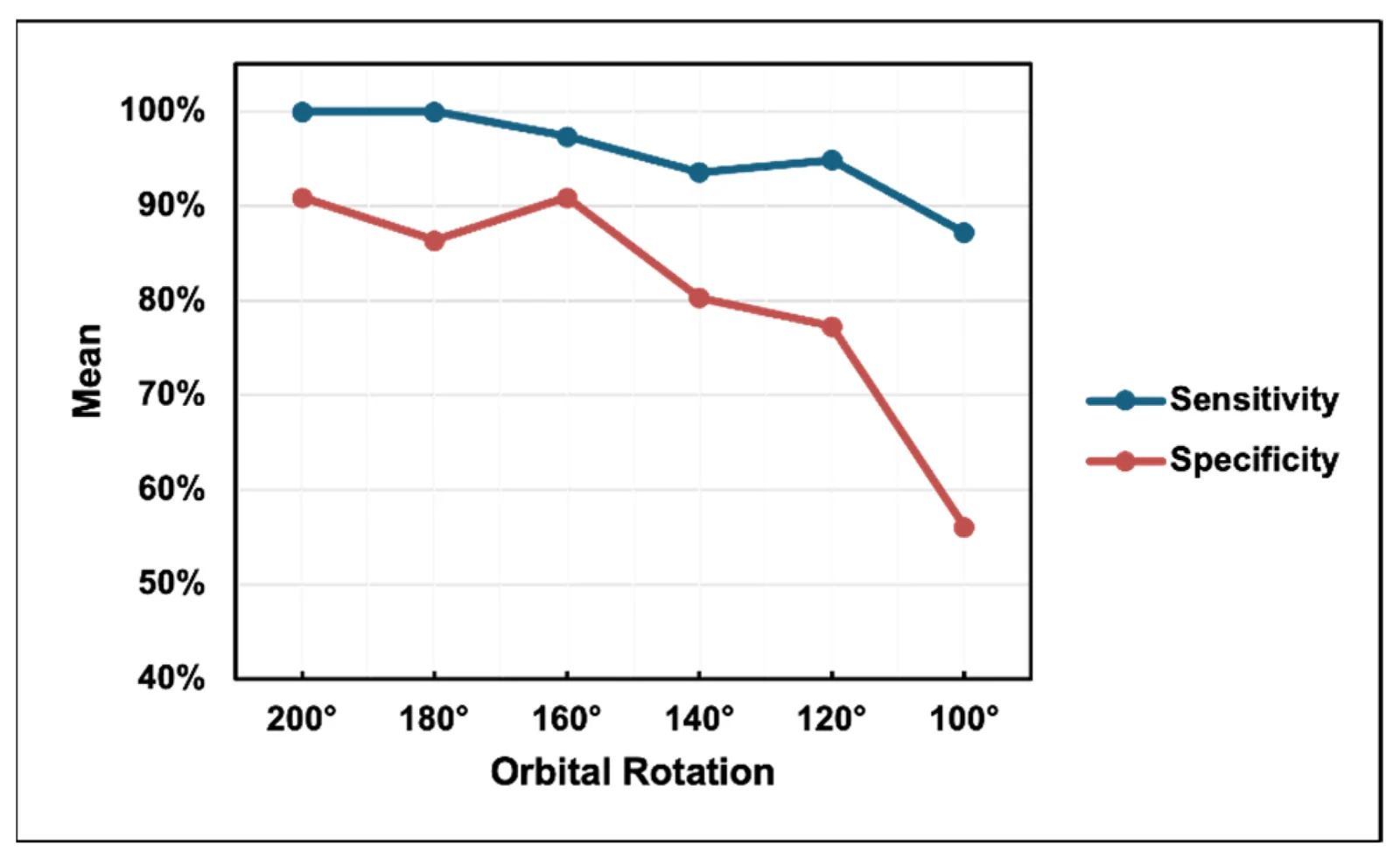
Sensitivity and Specificity for detection of intra-articular screw position vs. Orbital rotation.

**Figure 6:**
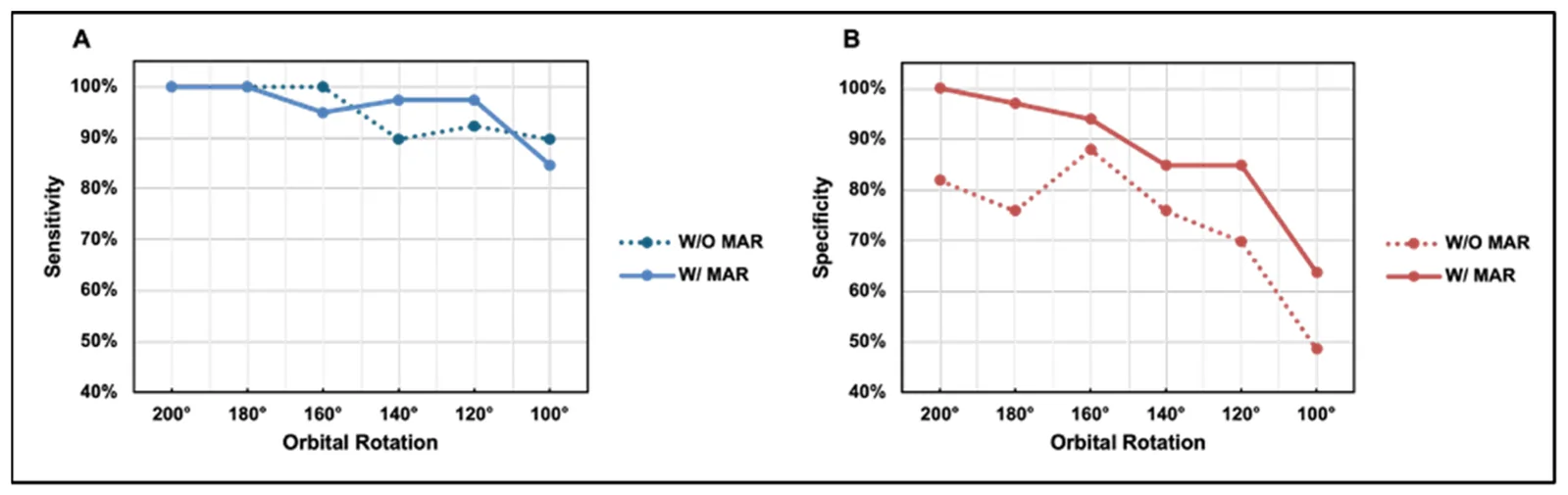
Sensitivity (**A**) and Specificity (**B**) for detection of intra-articular screw position ± MAR vs. Orbital Rotation.

**Figure 7:**
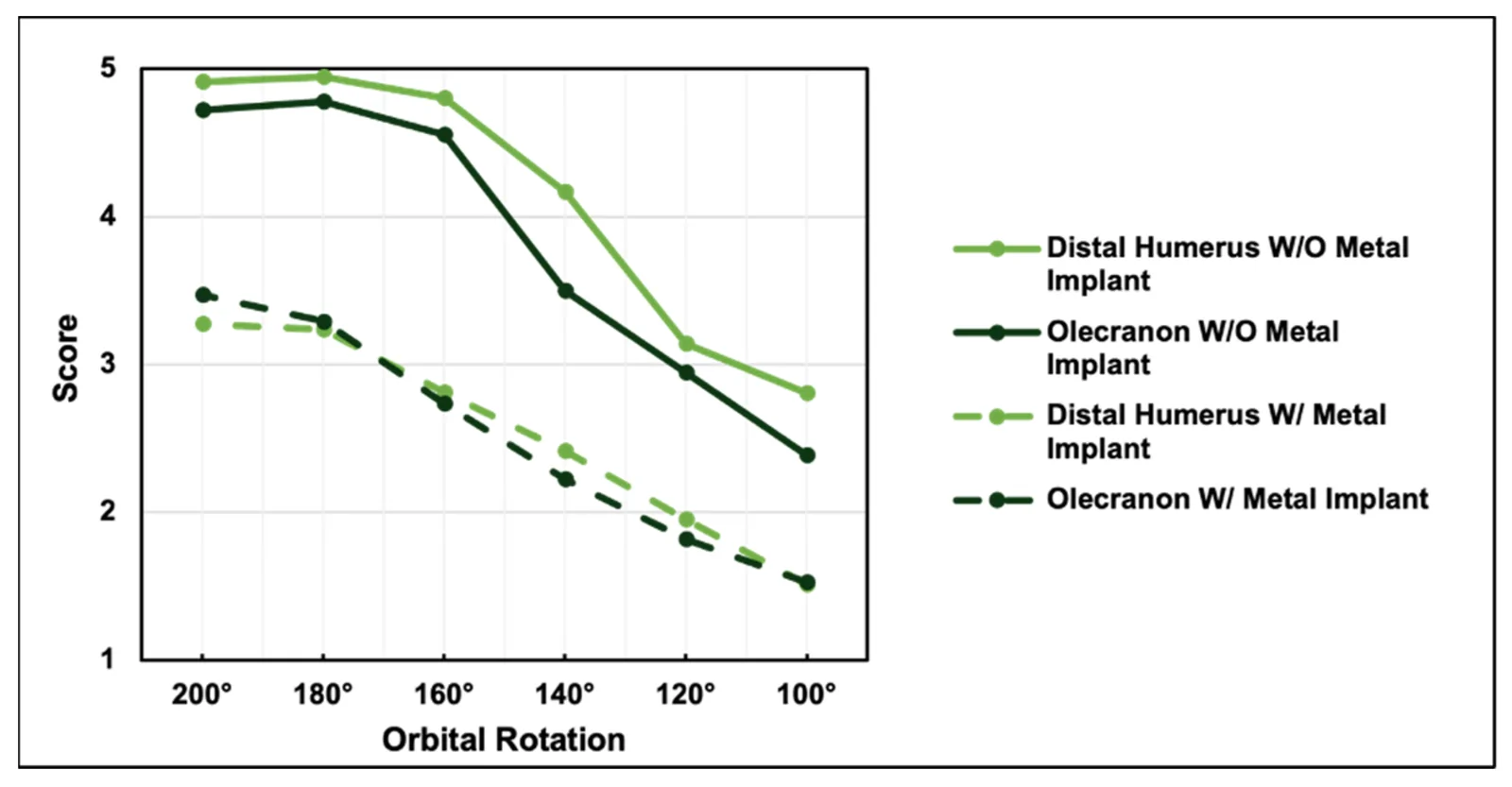
Assessment of Articular Surface vs. Orbital rotation.

**Figure 8:**
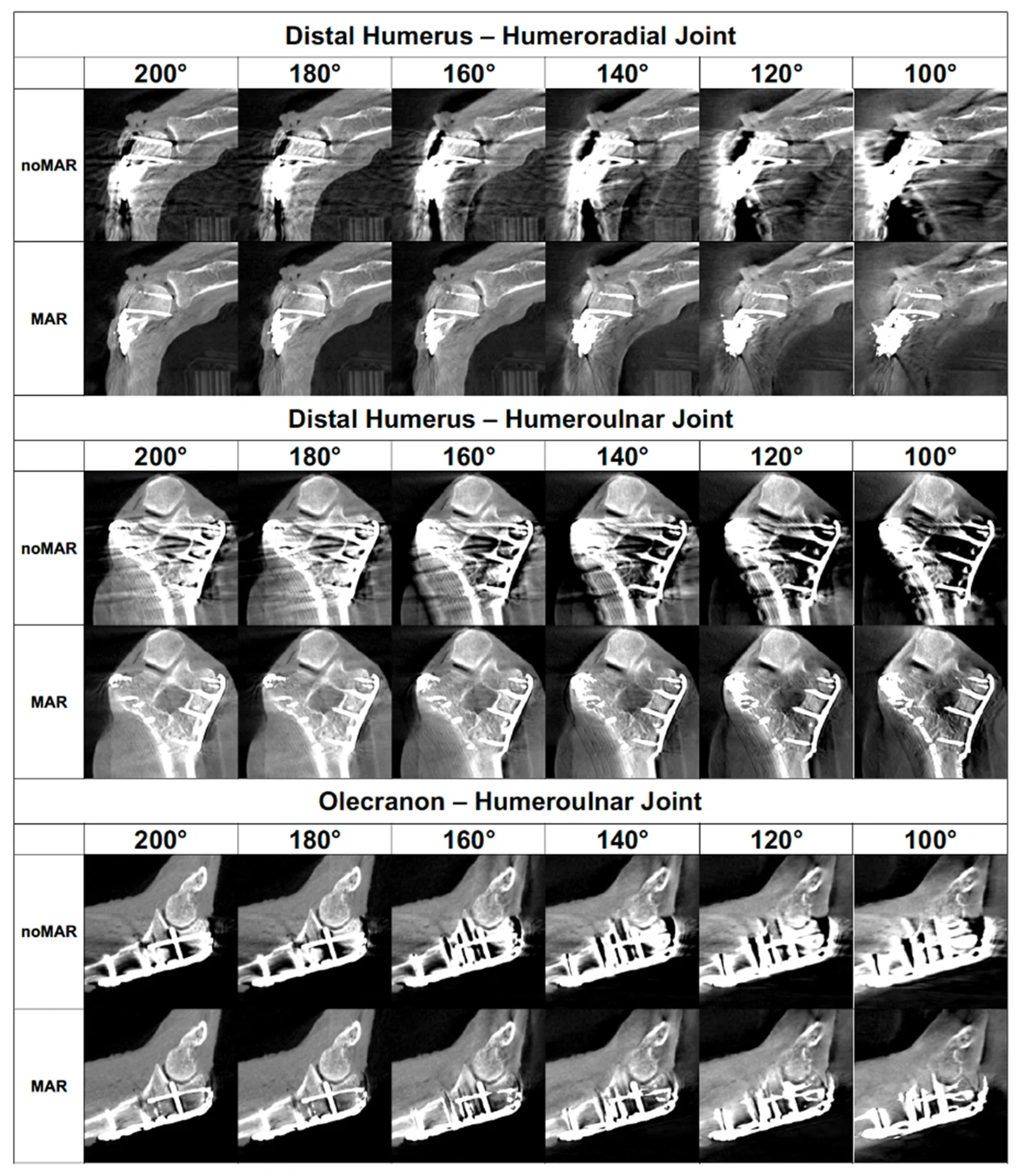
Visual examples.

**Table 1:** Reduction in orbital rotation.

Orbital Rotation	200°	180°	160°	140°	120°	100°
Start Position	100°	100°	100°	90°	80°	70°
End Position	−100°	−80°	−60°	−50°	−40°	−30°
Number of Images	400	360	320	280	240	200
	Asymmetric Reduction			Symmetric Reduction		

**Table 2:** Breakdown of all 3D scans.

	Cadavers	Arms	Anatomy	Scans	Orbital Rotation	Total
***n***	3	6	2	5	6	360
Details			Distal Humerus Olecranon	W/O Metal	200°	
				Correct Screw	180°	
				W/Metal	160°	
				W/Metal + MAR	140°	
				Incorrect Screw	120°	
				W/Metal	100°	
				W/Metal + MAR		

**Table 3:** Friedman test and post hoc cut-off in the assessment of the articular surface.

Assessment Articular Surface vs. Orbital Rotation	Friedman-Test *p*	Orbital Rotation Cut-Off (*p* ≥ 0.05)
Olecranon W/O Metal Implant	<0.001	160°
Olecranon W/Metal Implant	<0.001	180°
Distal Humerus W/O Metal Implant	<0.001	160°
Distal Humerus W/Metal Implant	<0.001	180°

## Data Availability

The raw data supporting the conclusions of this article will be made available by the authors upon request.

## Abbreviations

The following abbreviations are used in this manuscript:

CBCT	cone-beam computed tomography
ICC	intraclass correlation coefficient
IQ	image quality
MAR	metal artifact reduction

## Appendix A

**Table A1:** Questionnaire for the Assessment of Image Quality and Screw Position.

Score	Subjective Image Quality	Cortical Bone Visualization	Trabecular Bone Visualization	Articular Surface Visualization	Artifacts	Overall Clinical Assessment	Intra-Articular Screw Position	Certainty in the Assessment
**1**	Reduced	Not visible	Not visible	Not visible	Very disturbing	Not possible		Very low
**2**	Slightly reduced	Barely visible	Barely visible	Barely visible	Disturbing	Limited		Low
**3**	Acceptable	Acceptable	Acceptable	Acceptable	Moderate	Possible		Moderate
**4**	Good	Good	Good	Good	Few	Good		High
**5**	Very good	Very good	Very good	Very good	None	Very good		Very high
**0/1**							No/Yes	

**Table A2:** Sensitivity and specificity for intra-articular screw detection relative to the 200° imaging-based reference condition according to orbital rotation and reconstruction condition.

Orbital Rotation	Reconstruction	TP *	FN *	Sensitivity, % (95% CI)	TN *	FP *	Specificity, % (95% CI)
100°	Standard	35	4	89.7 (75.8–97.1)	16	17	48.5 (30.8–66.5)
100°	MAR	33	6	84.6 (69.5–94.1)	21	12	63.6 (45.1–79.6)
120°	Standard	36	3	92.3 (79.1–98.4)	23	10	69.7 (51.3–84.4)
120°	MAR	38	1	97.4 (86.5–99.9)	28	5	84.8 (68.1–94.9)
140°	Standard	35	4	89.7 (75.8–97.1)	25	8	75.8 (57.7–88.9)
140°	MAR	38	1	97.4 (86.5–99.9)	28	5	84.8 (68.1–94.9)
160°	Standard	39	0	100.0 (91.0–100.0)	29	4	87.9 (71.8–96.6)
160°	MAR	37	2	94.9 (82.7–99.4)	31	2	93.9 (79.8–99.3)
180°	Standard	39	0	100.0 (91.0–100.0)	25	8	75.8 (57.7–88.9)
180°	MAR	39	0	100.0 (91.0–100.0)	32	1	97.0 (84.2–99.9)
200°	Standard	39	0	100.0 (91.0–100.0)	27	6	81.8 (64.5–93.0)
200°	MAR	39	0	100.0 (91.0–100.0)	33	0	100.0 (89.4–100.0)

* FN, false negative; FP, false positive; MAR, metal artifact reduction; TN, true negative; TP, true positive.
